# Sensitization of neurons in the central nucleus of the amygdala via the decreased GABAergic inhibition contributes to the development of neuropathic pain-related anxiety-like behaviors in rats

**DOI:** 10.1186/s13041-014-0072-z

**Published:** 2014-10-04

**Authors:** Hong Jiang, Dong Fang, Ling-Yu Kong, Zi-Run Jin, Jie Cai, Xue-Jing Kang, You Wan, Guo-Gang Xing

**Affiliations:** Neuroscience Research Institute, Peking University, 38 Xue-Yuan Road, Beijing, 100191 P.R. China; Department of Neurobiology, School of Basic Medical Sciences, Peking University Health Science Center, Beijing, 100191 P.R. China; Key Laboratory for Neuroscience, Ministry of Education and Ministry of Health, Beijing, 100191 P.R. China

**Keywords:** Anxiety, Neuropathic pain, Firing pattern, CeA, GABA

## Abstract

**Background:**

Despite high prevalence of anxiety accompanying with chronic pain, the mechanisms underlying pain-related anxiety are largely unknown. With its well-documented role in pain and emotion processing, the amygdala may act as a key player in pathogenesis of neuropathic pain-related anxiety. Pain-related plasticity and sensitization of CeA (central nucleus of the amygdala) neurons have been shown in several models of chronic pain. In addition, firing pattern of neurons with spike output can powerfully affect functional output of the brain nucleus, and GABAergic neurons are crucial in the modulation of neuronal excitability. In this study, we first investigated whether pain-related plasticity (e.g. alteration of neuronal firing patterns) and sensitization of CeA neurons contribute to nerve injury-evoked anxiety in neuropathic rats. Furthermore, we explored whether GABAergic disinhibition is responsible for regulating firing patterns and intrinsic excitabilities of CeA neurons as well as for pain-related anxiety in neuropathic rats.

**Results:**

We discovered that spinal nerve ligation (SNL) produced neuropathic pain-related anxiety-like behaviors in rats, which could be specifically inhibited by intra-CeA administration of anti-anxiety drug diazepam. Moreover, we found potentiated plasticity and sensitization of CeA neurons in SNL-induced anxiety rats, of which including: 1) increased burst firing pattern and early-adapting firing pattern; 2) increased spike frequency and intrinsic excitability; 3) increased amplitude of both after-depolarized-potential (ADP) and sub-threshold membrane potential oscillation. In addition, we observed a remarkable reduction of GABAergic inhibition in CeA neurons in SNL-induced anxiety rats, which was proved to be important for altered firing patterns and hyperexcitability of CeA neurons, thereby greatly contributing to the development of neuropathic pain-related anxiety. Accordantly, activation of GABAergic inhibition by intra-CeA administration of muscimol, a selective GABA_A_ receptors agonist, could inhibit SNL-induced anxiety-like behaviors in neuropathic rats. By contrast, suppression of GABAergic inhibition by intra-CeA administration of bicuculline, a selective GABA_A_ receptors antagonist, produced anxiety-like behavior in normal rats.

**Conclusions:**

This study suggests that reduction of GABAergic inhibition may be responsible for potentiated plasticity and sensitization of CeA neurons, which likely underlie the enhanced output of amygdala and neuropathic pain-related anxiety in SNL rats.

**Electronic supplementary material:**

The online version of this article (doi:10.1186/s13041-014-0072-z) contains supplementary material, which is available to authorized users.

## Background

Chronic pain is a multidimensional experience including sensory, affective and cognitive components [1]. A large number of chronic pain patients suffer from comorbidities such as anxiety, depression, and sleep disturbance [2,3]. Despite high prevalence of anxiety accompanying with neuropathic pain, the molecular and cellular mechanisms underlying neuropathic pain-related anxiety are largely unknown.

With its well-documented role in pain and emotion processing [4-6], the amygdala may act as a key player in pathogenesis of neuropathic pain-related anxiety following nerve injury. The amygdala is composed of functionally and morphologically heterogeneous subnuclei with complex interconnectivity [7,8]. The lateral/basal lateral amygdala (LA/BLA) is conceptualized as the main input of the amygdala, and the central nucleus of the amygdala (CeA) as the main output station [7-9]. Although amygdala circuitry in conditioned fear is widely described [10-12], the causal underpinning of pain-related anxiety has not yet been explored.

Pain-related plasticity and sensitization of CeA neurons has been shown in several models of chronic pain [6,13-15], we speculated that this kind of plasticity and sensitization probably contributes to the development of nerve injury-evoked anxiety in neuropathic pain rats. In addition, as the major output nucleus of the amygdala, CeA mediates autonomic and behavioral responses associated with fear and anxiety via projections to the brain stem [16,17]. Firing pattern of neurons with spike output can powerfully affect functional output of the brain nucleus [18-20]. Therefore, alteration of firing patterns in CeA neurons will affect amygdala output and finally regulate the affective component of neuropathic pain. It is well accepted that the amygdala is responsible for the regulation of emotional behaviors such as depression and anxiety [21-23]. We here inferred that CeA output to other brain areas would control the anxiety processing, which probably underlies the pain-related anxiety after nerve injury. However, what is the exact classification of firing patterns in spontaneous/evoked activities and the physiological significance of such firing patterns of CeA neurons still remains largely unclear.

Both increase in excitatory neuron and decrease in inhibitory neuron equally contribute to the hyperexcitability of CeA nucleus. Since CeA is under strong local inhibitory control (large population of GABAergic neurons) [11], the role of GABAergic neurons would be a determinant contributor for excitability of CeA nucleus. Using optogenetic technique, specific activation of GABAergic neurons is reported to induce brief action potential (AP) discharges in CA3 pyramidal neurons, followed by prolonged suppression of ongoing epileptiform activity during light exposure [24]. In addition, GABAergic inhibition has been shown to play a crucial role in generation of rhythmic activity and network oscillation [25,26]. These findings strongly support the role of GABAergic neurons in the modulation of neuronal excitability [27,28]. We thus hypothesized that loss or reduction of GABAergic inhibition would probably underlie the sensitization of CeA neurons in neuropathic rats.

In this study, we first investigated whether pain-related plasticity (e.g. alteration of neuronal firing patterns) and sensitization of CeA neurons contribute to nerve injury-evoked anxiety in neuropathic rats. Furthermore, we explored whether GABAergic inhibition is responsible for regulating firing patterns and intrinsic excitabilities of CeA neurons as well as for pain-related anxiety in neuropathic rats. We suggest that reduction of GABAergic inhibition may be responsible for potentiated plasticity and sensitization of CeA neurons, which likely underlie the enhanced output of amygdala and neuropathic pain-related anxiety in SNL rats.

## Results

### Anxiety-like behaviors in rats with SNL-induced neuropathic pain

**Figure 1:**
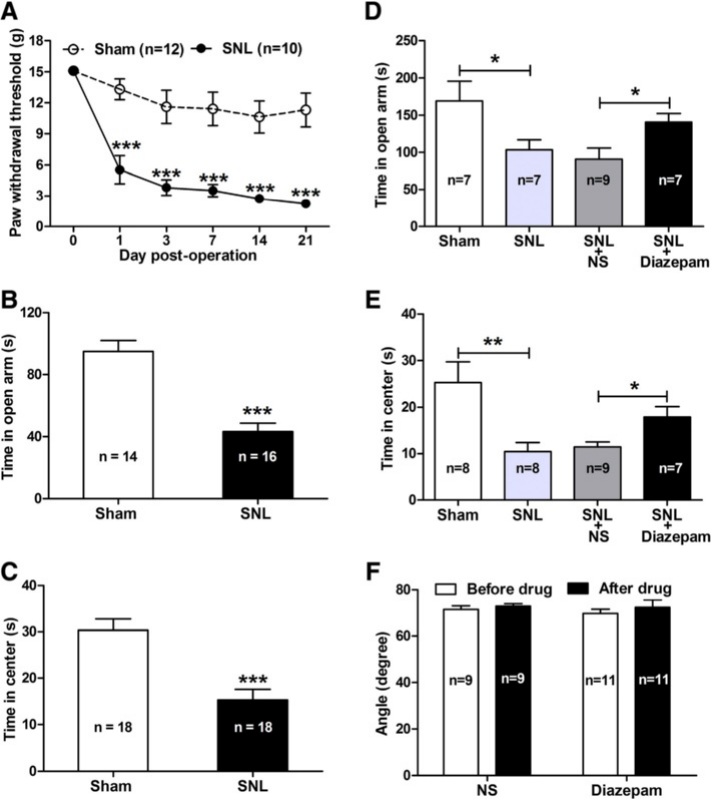
**Spinal nerve ligation (SNL) induces mechanical allodynia and anxiety-like behaviors in rats. (A − C)**: pain behavior and anxiety-like behaviors. Note that SNL produces obvious pain behavior as measured by 50% paw withdrawal threshold (PWT) (**A**, ***p < 0.001, two-way ANOVA, n = 10 SNL, 12 sham) and anxiety-like behaviors as measured by spent time in elevated plus-maze (EPM) (**B**, ***p < 0.001, SNL *versus* sham, two-tailed unpaired t-test, n = 10 SNL, 12 sham) and open-field test (**C**, ***p < 0.001, SNL *versus* sham, two-tailed unpaired t-test, n = 18/group). **(D − F)**: effects of intra-CeA administration of diazepam on anxiety-like behaviors and locomotor function in SNL rats. Note that diazepam (2 μg/μl) dramatically inhibits SNL-induced anxiety-like behaviors as measured by EPM test **(D)** and open-field test **(E)** and does not affect the locomotor function of rats as measured by inclined-plate test **(F)** (*p < 0.05, **p < 0.01, one-way ANOVA, n = 7 − 11/group).

As shown in Figure 1, ligation of the lumbar 5 spinal nerve in rats produced significant mechanical allodynia that is associated with anxiety-like behaviors. As a behavioral sign of pain allodynia, ipsilateral paw withdrawal threshold (PWT) responding to von Frey filaments was significantly decreased in spinal nerve ligated (SNL) rats compared to sham-operated rats (p < 0.001, Figure 1A) from day 1 to day 21 following nerve injury. Meanwhile, SNL also produced obvious anxiety-like behaviors in rats exhibiting neuropathic pain (Figure 1B and C). Anxiety-like behaviors were evaluated by elevated plus-maze (EPM) test and open-field test at 10 days after SNL when stable pain hypersensitivity emerged in rats. In elevated plus-maze test, SNL rats spent less time in open arms compared to sham-operated rats (SNL 43.31 ± 5.53 sec *versus* sham 94.92 ± 7.14 sec, p < 0.001, Figure 1B); while in open-field test, the time spent in the central zone in SNL rats was also decreased significantly (SNL 15.28 ± 2.29 sec *versus* sham 30.28 ± 2.42 sec, p < 0.001, Figure 1C). These data suggest that spinal nerve injury induces both mechanical allodynia and anxiety-like behaviors in neuropathic rats.

Moreover, by intra-CeA administration of diazepam, a classical anti-anxiety drug that has been widely used in clinic [29], we discovered that diazepam (2 μg/μl) remarkably inhibited the SNL-induced anxiety-like behaviors but did not affect pain behaviors (Figure 1D **−** F; Additional file 1: Figure S1) in neuropathic rats. The SNL-induced reduction of the time spent in open arms was statistically rescued by treatment of diazepam in contrast to normal saline (NS) (Diazepam 140.4 ± 11.72 sec *versus* NS 91.00 ± 14.70 sec, p < 0.05, Figure 1D). Similarly, the decreased time spent in center of open-field test in SNL rats was also restored by treatment of diazepam compared to NS (Diazepam 17.88 ± 2.24 sec *versus* NS 11.42 ± 1.08 sec, p < 0.05, Figure 1E). As our expectation, intra-CeA injection of diazepam at the same dose had no significant effect on both mechanical allodynia (p > 0.05, in contrast to NS and pre-drug) (Additional file 1: Figure S1) and locomotor function (p > 0.05, in contrast to NS and pre-drug, respectively, Figure 1F) in SNL rats. These results indicate that nerve injury induces anxiety-like behaviors in neuropathic rats, which are specifically sensitive to anti-anxiety drugs. Additionally, the CeA plays an important role in the development of nerve injury-evoked anxiety-like behaviors.

### Changes in firing pattern of the CeA neurons in SNL-induced anxiety rats

**Figure 2:**
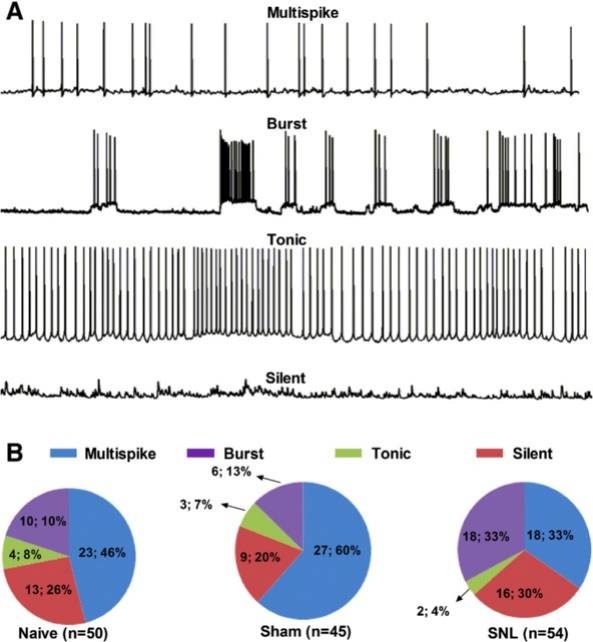
**Alteration of spontaneous firing patterns in CeA neurons in SNL-induced anxiety rats. (A)**: representative of four different firing patterns of CeA neurons recorded in spontaneous discharges mode under whole-cell current-clamp recording. **(B)**: proportions of four firing patterns in the CeA neurons in naïve, sham and SNL rats, respectively. Note that the proportion of burst firing is increased significantly in SNL rats compared to naïve and sham rats. However, the proportion of multispike and tonic firing neurons are decreased relatively compared to naïve and sham rats. N = 50 cells from 7 naïve rats, 45 cells from 6 sham rats, and 54 cells from 8 SNL rats, respectively.

In order to determine whether excitability of CeA neurons was increased in SNL-induced anxiety rats, we first investigated the electrophysiological characteristics of CeA neurons in naïve rats. A total of 52 neurons were recorded from the amygdala slices in the present study. Four patterns of the CeA neurons discharges were observed in spontaneous-firing recording mode during a 60-sec period from all recorded cells. They were multispike firing (neurons also called “irregular firing”)-, burst firing (neurons with recurrent, abrupt high frequency firing featured with depolarized membrane potential)-, tonic firing (neurons with regular, high frequency and nonstop firing)-, and silent firing (neurons unable to fire in spontaneous state)-neurons (Figure 2A). Among spontaneous-firing modes, the pattern of burst firing, which generates high frequency output of an entire regional population, is predicted to promote the synchronization between interconnected loci in central nervous system (CNS) networks [30] and contribute to normal non-REM sleep and some types of epileptic seizures. Hence, once burst firing is out of phase, which would probably contribute to some pathological diseases such as epilepsy, anxiety and sleep disorders. In this study, we found that the pattern of burst firing was significantly increased in SNL-induced anxiety rats (naïve 10%, sham 13% *versus* SNL 33%, respectively) (Figure 2B). In contrast, the pattern of tonic firing was comparatively decreased (naïve 8%, sham 7% *versus* SNL 4%, respectively) (Figure 2B). Thus, increased pattern of burst firing as an important feature of epileptiform activities and tonic excitation emerges in SNL-induced anxiety state.

**Figure 3:**
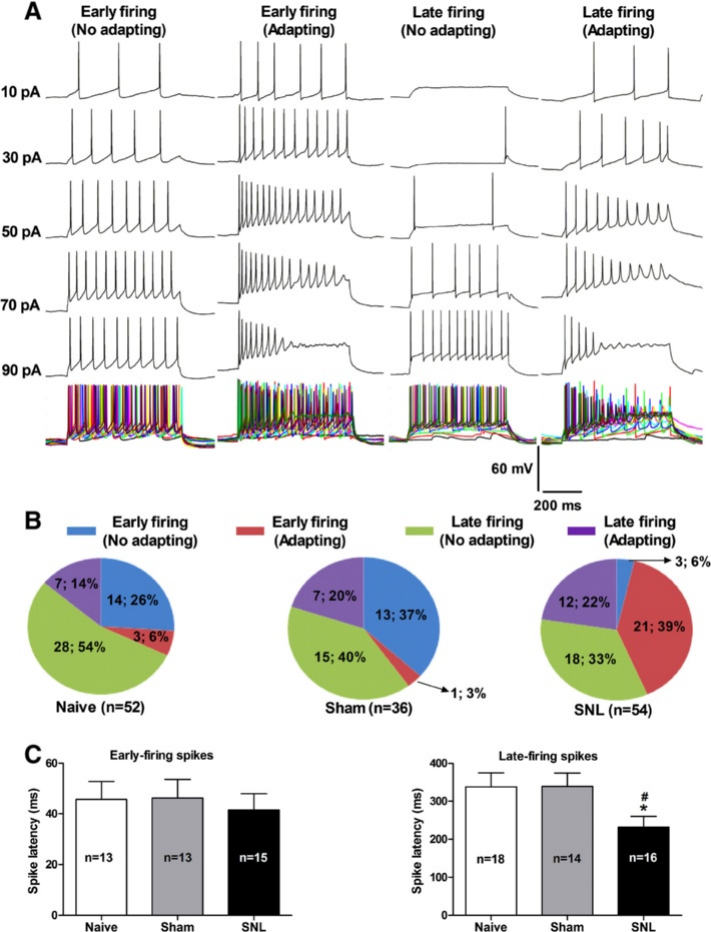
**Alteration of evoked firing patterns and reduction of spike latency in CeA neurons in SNL-induced anxiety rats. (A)**: representative of four different firing patterns of CeA neurons recorded in evoked discharges mode under whole-cell current-clamp recording. Action potentials are evoked by a series of 600-ms depolarizing current pulses in 10 pA increment from 0 pA to 110 pA. **(B)**: proportions of four firing patterns in the CeA neurons in naïve, sham and SNL rats, respectively. Note that the proportion of early firing-adapting pattern in CeA neurons is increased significantly in SNL rats compared to naïve and sham rats. N = 52 cells from 7 naïve rats, 36 cells from 6 sham rats, and 54 cells from 8 SNL rats, respectively. **(C)**: summaries of spike latency in early-firing and late-firing pattern in CeA neurons in naïve, sham and SNL rats. Note that the spike latency is decreased remarkably in late-firing neurons but not in early-firing neurons in SNL rats compared to naïve and sham rats, ^#^p < 0.05, compared to naïve; *p < 0.05, compared to sham, one-way ANOVA, n = 14 − 18/group.

Apart from spontaneous firing, the depolarizing current-evoked firing patterns of action potentials were also examined in the same neurons. A series of step currents (duration 600 ms, increment 10 pA) from 10 pA to 110 pA were applied to the recorded neuron. According to spike latency and adapting features, all recorded neurons were initially classified as four firing patterns: 1) Early firing and no adapting, which took a short latency (less than 100 ms) to evoke the 1^st^ spike, and meanwhile, along with increased current injection, the spike fired regularly; 2) Early firing and adapting, similar to pattern 1, which took a short latency but the spike firing displayed accommodatingly; 3) Late firing and no adapting, which took a long latency (more than 100 ms and less than 600 ms) to evoke the 1^st^ spike, and the spike fired regularly along with increasing current injection; 4) Late firing and adapting, which took a long latency like pattern 3, but the spike firing displayed accommodatingly (Figure 3A). In contrast to naïve and sham rats, the percentage of early firing with adapting (pattern 2) neurons was markedly increased in SNL-induced anxiety rats (naïve 6%, sham 3% *versus* SNL 39%, respectively) (Figure 3B), whereas the latency of late firing with or without adapting (pattern 3 and pattern 4) neurons in SNL rats was decreased prominently (naïve 338.3 ± 36.6 ms, sham 339.5 ± 34.7 ms *versus* SNL 231.8 ± 28.3, p < 0.05, respectively) (Figure 3C). Thus, those increased early firing pattern and spike adapting as well as decreased spike latency indicate that nerve injury induces hyperexcitability in CeA neurons.

### Increases in amplitude and duration of after depolarized potential (ADP) as well as in amplitude of membrane potential oscillation in SNL-induced anxiety rats

**Figure 4:**
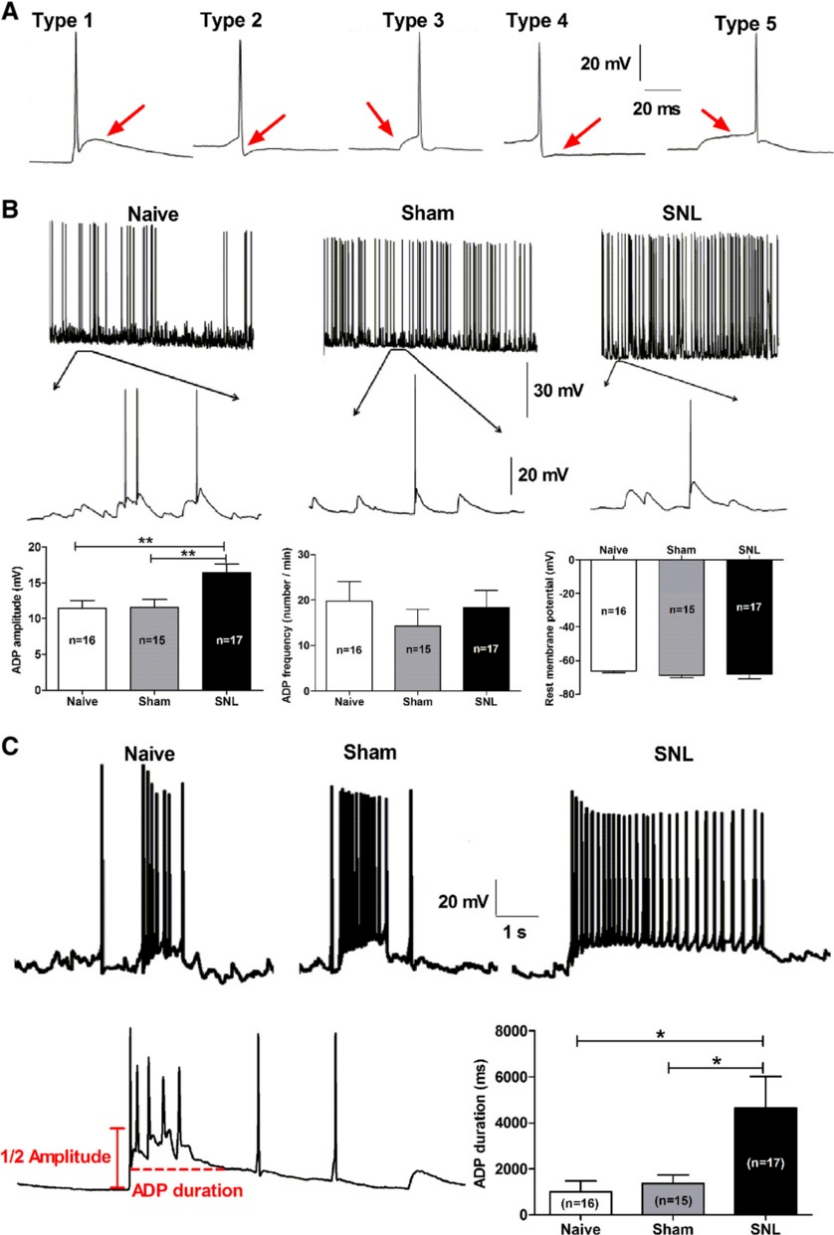
**Increases in amplitude and duration of after depolarized potential (ADP) in CeA neurons in SNL-induced anxiety rats. (A)**: representative of five spontaneous action potential types (type 1 to type 5) of CeA neurons under whole-cell current-clamp recording. Red arrows show the main features of each action potential type. Detailed description is mentioned in the text. **(B and C)**: summaries of amplitude and frequency of ADP and rest membrane potential (RMP) of the CeA neurons in naïve, sham and SNL rats. **(B)**: examples represent characteristics of ADP in multispike firing neurons exhibiting type 1 action potentials. Lower panel in each group: enlarged trace showing ADP of type 1 neurons. Summaries of the amplitude and frequency of ADP and RMP of type 1 neurons among naïve, sham and SNL groups are displayed below the representative of spikes. **(C)**: examples represent characteristics of ADP in burst-firing neurons. Lower panel: enlarged trace showing the measurement of ADP amplitude and duration. Summary of ADP duration of burst-firing neurons among three groups is displayed in the right bottom. Note that SNL induces a significant increase in both amplitude (**B**-left bottom, **p < 0.01, one-way ANOVA) and duration (**C**-right bottom, *p < 0.05, one-way ANOVA) but not frequency (**B**-middle, p > 0.05, one-way ANOVA) of ADP in CeA neurons. Moreover, no significant difference is observed on RMP among naïve, sham and SNL groups (**B**-right bottom, p > 0.05, one-way ANOVA). N = 16 cells from 4 naïve rats, 15 cells from 5 sham rats and 17 cells from 6 SNL rats, respectively.

Under spontaneous recording, multiple types of action potentials (APs) were observed in CeA neurons. For instance, type 1 neurons were featured with obvious “after depolarized potential (ADP)”, and this type accounted for ~40% of total neurons; type 2 neurons, which accounted for ~8% of total neurons, displayed apparent fast “after hyperpolarized potential (AHP)”; type 3 neurons were marked with abrupt depolarization before AP threshold and counted ~8% of total neurons; while type 4 neurons, which accounted for ~10% of total neurons, were featured with slow AHP that took a long time to return baseline; and type 5 neurons, which accounted for ~4% of total neurons, were with characteristic of long but slow depolarization before reaching AP threshold as compared to type 3 neurons (Figure 4A). Because type 1 neurons were the main component in total neurons, we thus analyzed their electrophysiological properties in the following study.

It is well accepted that ADP is an important factor for generation of burst firing [31-33], we therefore investigated whether the ADP features including amplitude and duration were altered in SNL-induced anxiety rats. The difference of membrane potential between the peak of ADP and the baseline was measured as the ADP amplitude (as shown in Figure 4C). We found that SNL induced a significant increase in amplitude (SNL 16.44 ± 1.17 mV *versus* naïve 11.46 ± 1.11 mV and sham 11.58 ± 1.10 mV, p < 0.01) but not frequency (numbers per 1 min) of the ADP (Figure 4B). Unexpectedly, although rest membrane potential (RMP) is closely related to ADP amplitude [34], we did not observe any significant difference on RMP among naïve, sham and SNL groups (p > 0.05). Similar as the ADP amplitude, the duration of ADP was also increased significantly in SNL rats (4652 ± 1372 ms) as compared with that in naïve (1001 ± 488 ms) and sham (1381 ± 364 ms) rats (p < 0.05, Figure 4C).

**Figure 5:**
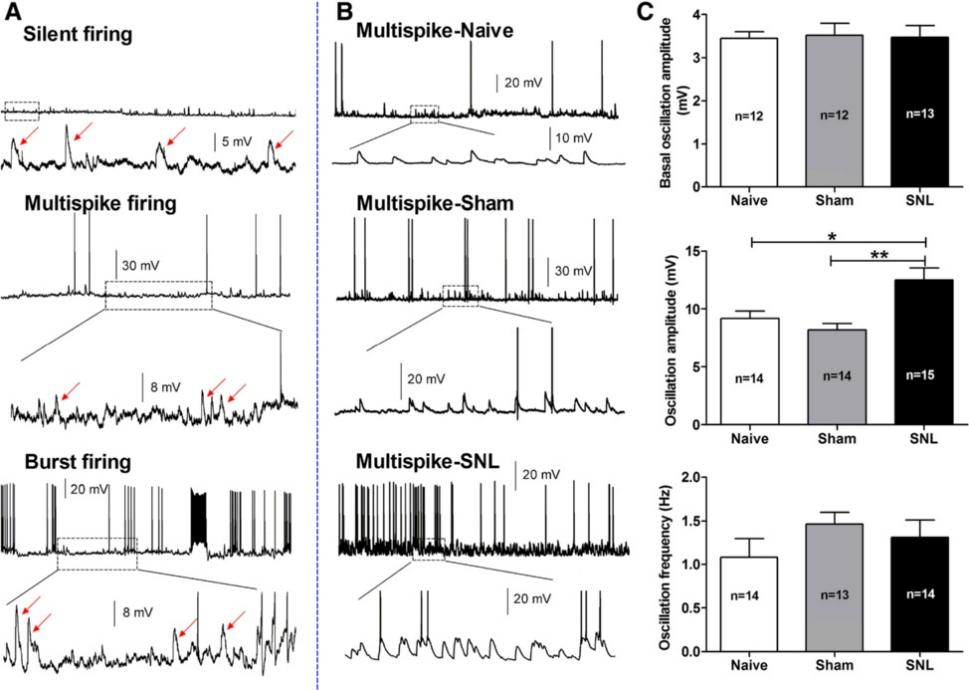
**Increase in amplitude of membrane oscillation in CeA neurons in SNL-induced anxiety rats. (A)**: representative of three different firing patterns and membrane oscillation of CeA neurons recorded in spontaneous discharges mode under whole-cell current-clamp recording. Lower panel in each firing pattern: enlarged trace shows that membrane oscillation emerges in silent, multispike and burst firing neurons in naïve rats. **(B)**: representative multispike firing pattern recorded from CeA neurons in naïve, sham and SNL rats. Lower panel in each group: enlarged trace showing enhanced membrane potential oscillation in SNL rats compared to naïve and sham rats. **(C)**: summary of the amplitude and frequency of membrane oscillation in CeA neurons in naïve, sham and SNL rats. Note that although no difference was found in amplitude of basal oscillation among three groups (upper), the amplitude (middle) but not the frequency (lower) of main oscillation is increased dramatically in SNL rats compared to naïve and sham rats. *p < 0.05, **p < 0.01, one-way ANOVA, n = 12 − 15/group. All recorded cells in each group are obtained from animals of naïve (4 rats); sham (4 rats) and SNL (6 rats), respectively.

In addition, oscillation of membrane potential is another key factor for the occurrence of burst firing. High frequency burst firing is resulted from a high proportion of oscillation sinusoid of membrane potential when it reaches the AP threshold [35]. The amplitude and frequency of sub-threshold oscillation in membrane potential are quite important for excitability of cells. In this study, we found that the sub-threshold membrane potential oscillation widely existed in silent, multispike and burst firing pattern (Figure 5A). Although no difference was found in amplitude of basal oscillation (defined as amplitude less than 5 mV) among naïve, sham and SNL groups (Figure 5C, upper lane), the amplitude (Figure 5C, middle lane) but not the frequency (Hz, Figure 5C, lower lane) of main oscillation (defined as amplitude more than 5 mV) was increased dramatically in SNL rats (12.51 ± 1.04 mV) compared to naïve (9.18 ± 0.66 mV, p < 0.05) and sham (8.20 ± 0.54 mV, p < 0.01) rats. Representative multispike firing recorded from CeA neurons in naïve, sham and SNL rats are shown in Figure 5B, lower panel: enlarged trace showing enhanced membrane potential oscillation in SNL rats compared to naïve and sham rats.

### Increases in spike frequency and adapting firing ratio in CeA neurons evoked by a 300 pA-depolarizing current in SNL-induced anxiety rats

**Figure 6:**
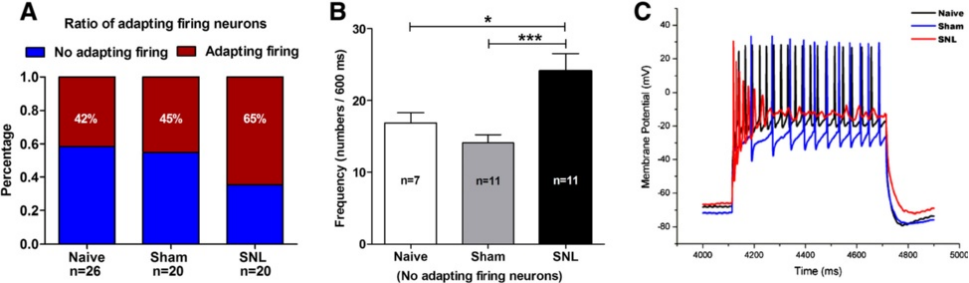
**Increase in both percentage of adapting firing neurons and frequency of no adapting firing neurons in CeA nucleus in SNL-induced anxiety rats. (A)**: percentage of adapting (red color) and no adapting (blue color) firing neurons. Note that the percentage of adapting firing neurons is significantly increased in SNL rats compared to naïve and sham rats. **(B)**: frequency of no adapting firing neurons. Note that the frequency of action potentials in no adapting firing neurons is prominently increased in SNL rats compared to naïve and sham rats. *p < 0.05, ***p < 0.001, one-way ANOVA, n = 7 naïve, 11 sham and 11 SNL. **(C)**: representative of evoked discharges of CeA neurons obtained from naïve, sham and SNL rats, respectively. Action potentials are evoked by a depolarizing current of 600 ms, 300 pA. Note that adapting firing pattern is shown in SNL rat, while no adapting firing pattern exhibits in naïve and sham rats. All recorded cells in each group are obtained from animals of naïve (3 rats); sham (5 rats) and SNL (6 rats), respectively.

In order to determine whether spike frequency was increased in SNL rats, a large depolarizing current of 600 ms, 300 pA was delivered to CeA neurons to ensure that all recorded cells could be evoked sufficient firing and the evoked discharges were elicited under an equal depolarizing current pulse. As shown in Figure 6, a 300 pA-depolarizing current intracellularly injected into CeA neurons could evoke two spike patterns, i.e. adapting and non-adapting firing. With respect to adapting neurons, we found that the percentage of this pattern neurons was much higher (65%) in SNL rats than that in naïve (42%) and sham (45%) rats (Figure 6A). With regard to non-adapting neurons, the frequency (spike numbers in 600 ms) was increased remarkably in SNL rats (24.11 ± 2.41) compared to naïve (16.86 ± 1.44, p < 0.05) and sham (14.09 ± 1.14, p < 0.001) rats (Figure 6B). The traces in Figure 6C represent the original discharges recorded from CeA neurons among three groups. Taken together, these data provide additional evidence showing that excitabilities of CeA neurons are significantly increased in SNL-induced anxiety rats.

### Changes in intrinsic membrane properties of CeA neurons in SNL-induced anxiety rats

**Figure 7:**
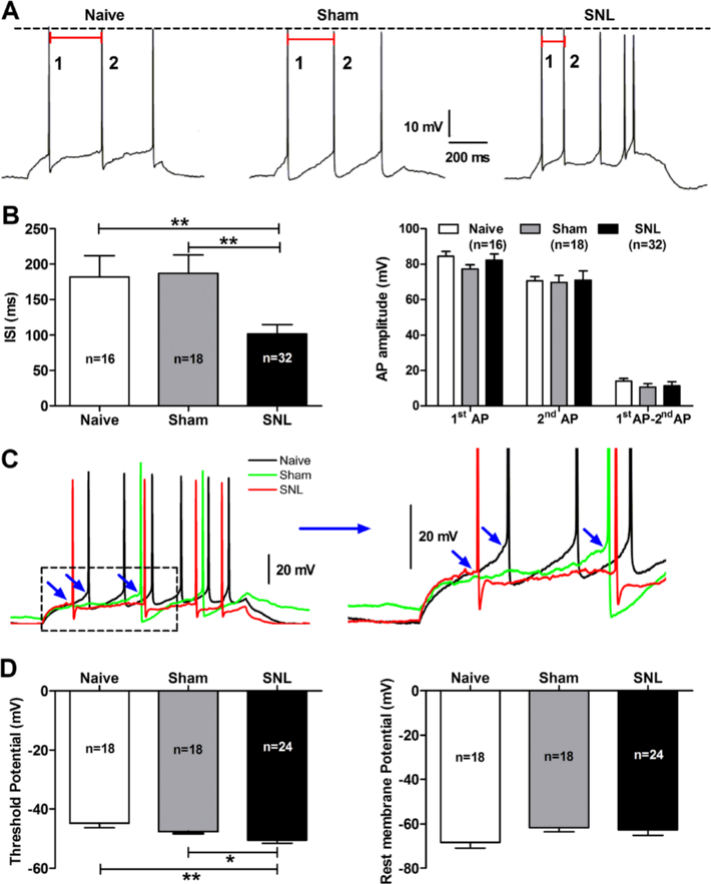
**Increase in intrinsic excitability of CeA neurons in SNL-induced anxiety rats. (A)**: representative of the 1^st^ and the 2^nd^ action potentials (APs) of CeA neurons evoked by a series of depolarizing step currents (duration 600 ms, increment 10 pA) from 0 pA to 110 pA. Red bar shows the time of inter-spike interval (ISI) between the 1^st^ AP and the 2^nd^ AP. **(B)**: summaries of ISI and amplitude of action potentials. Note that the ISI between the 1^st^ and the 2^nd^ AP is dramatically increased in SNL rats compared to naïve and sham rats. **p < 0.01, one-way ANOVA, n = 16 naïve, 18 sham and 32 SNL. No changes in amplitude of the 1^st^ and the 2^nd^ AP and the difference between amplitude of the 1^st^ and the 2^nd^ AP are found in SNL rats. **(C)**: examples represent threshold potential (TP) and rest membrane potential (RMP) of CeA neurons in naïve, sham and SNL rats, which are evoked by a series of depolarizing step currents (duration 600 ms, increment 10 pA) from 0 pA to 110 pA. Enlarged traces from the left dotted square are shown in the right panel. TP in each AP is showed by blue arrows. **(D)**: summaries of TP (left) and RMP (right) of CeA neurons among three groups. Note that the TP of CeA neurons is more hyperpolarized in SNL rats compared to naïve and sham rats. *p < 0.05, **p < 0.01, SNL *versus* naïve, one-way ANOVA, n = 16 naïve, 18 sham and 32 SNL. No difference is found in RMP of CeA neurons among three groups. All recorded cells in each group are obtained from animals of naïve (4 rats); sham (4 rats) and SNL (5 rats), respectively.

To further investigate whether the shift of firing pattern in SNL-induced anxiety rats was resulted from alteration of intrinsic electrogenic properties in CeA neurons, we examined the following parameters of CeA neurons, of which including: rest membrane potential (RMP), input resistance (R_in_), amplitude, rise and decay rate of action potentials as well as threshold potential and inter-spike interval (ISI) of action potentials. Here, action potentials were evoked by a series of depolarizing step currents (duration 600 ms, increment 10 pA) from 0 pA to 110 pA as described aforementioned. As shown in Figure 7, the time of ISI was significantly reduced in SNL rats (101.8 ± 13.09 ms) compared to naïve (181.8 ± 30.18 ms, p < 0.01) and sham (187.0 ± 25.81 ms, p < 0.01) rats (Figure 7A and B). In addition, the threshold potential was more hyperpolarized in SNL rats (−50.45 ± 1.08 mV) than that in naïve (−44.73 ± 1.55 mV, p < 0.01) and sham (−47.60 ± 0.63 mV, p < 0.05) rats (Figure 7C and D). However, no significant difference was observed on RMP, R_in_ and amplitude of action potentials as well as on rise and decay rate of the 1^st^ spike among three groups (Figure 7B and D; Additional file 2: Figure S2). Altogether, these data imply that the intrinsic excitabilities of CeA neurons are markedly increased in SNL rats, which likely underlie the development of SNL-induced anxiety-like behaviors.

### Reduction of GABAergic inhibition in CeA neurons in SNL-induced anxiety rats

**Figure 8:**
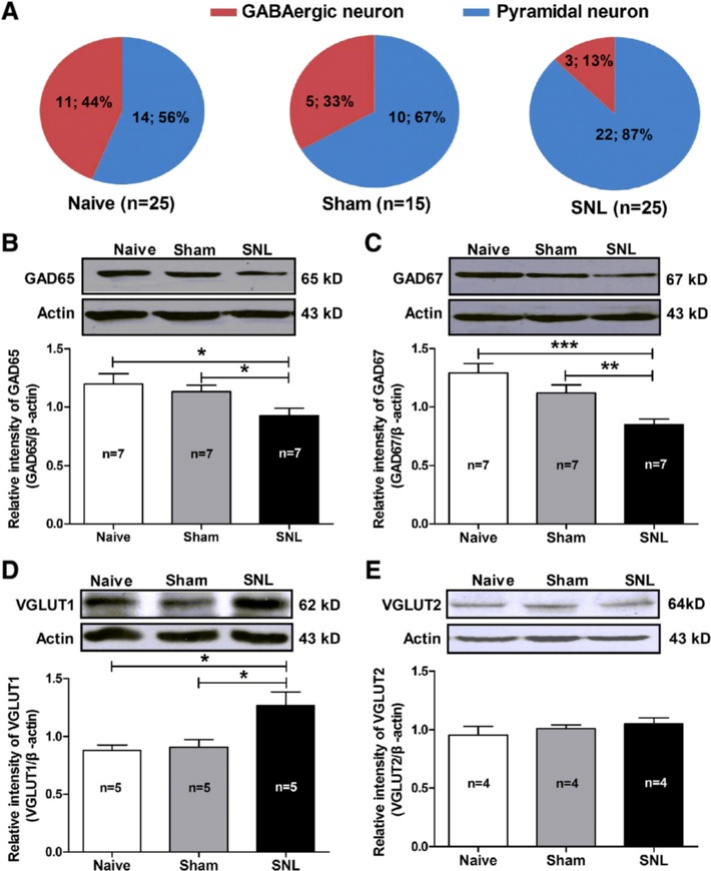
**Alteration of the proportion of GABAergic neurons and expression of GAD65, GAD67, VGLUT1 and VGLUT2 in the CeA in SNL-induced anxiety rats. (A)**: proportion of GABAergic neurons. Note that the proportion of GABAergic neurons in the CeA is decreased significantly in SNL rats compared to naïve and sham rats (n = 25 cells from 5 naïve rats, 15 cells from 4 sham rats and 25 cells from 5 SNL rats). **(B − C)**: Western blot detection of GAD65 and GAD67 expression in CeA nucleus. Upper: representative of Western blot bands; Lower: analysis of the relative intensity of GAD65 and GAD67. β-actin is used as an internal control. Note that the expression of both GAD65 **(B)** and GAD67 **(C)** are statistically decreased in SNL rats compared to naïve and sham rats. *p < 0.05, **p < 0.01, ***p < 0.001, one-way ANOVA, n = 7/group. **(D − E)**: Western blot assay of VGLUT1 and VGLUT2 expression in CeA nucleus. Upper: representative of Western blot bands; Lower: analysis of the relative intensity of VGLUT1 and VGLUT2. β-actin is used as an internal control. Note that expression of VGLUT1 but not VGLUT2 is increased significantly in SNL rats compared to naïve and sham rats. *p < 0.05, one-way ANOVA, n = 4 − 5/group.

Based on the aforementioned findings, we suggest that nerve injury induces enhancement of CeA output with burst firing and increased electrogenic features. As the main output of the amygdala, CeA is important for the modulation of emotion-related behaviors. Therefore, such enhancement of CeA output would probably contribute to the development of SNL-induced anxiety-like behaviors in neuropathic rats. However, the mechanism underlying the hyperexcitability of CeA neurons is largely unclear. In this study, we examined the proportion of GABAergic neurons in total recorded cells in the CeA slices. Firstly, using single-cell reverse-transcriptase PCR detection, we found that in naïve rats, GABAergic neurons occupied 44% of total recorded neurons; however, in SNL-induced anxiety rats, the percentage of GABAergic neurons reduced to 13% comparing to naïve and sham (33%) rats (Figure 8A). Single-cell reverse-transcriptase PCR detection of positive GABAergic and pyramidal neurons using the following markers such as GAD65 (glutamic acid decarboxylase-65) and GAD67 (glutamic acid decarboxylase-67) for GABAergic neuron, and VGLUT1 (vesicular glutamate transporter-1) and VGLUT2 (vesicular glutamate transporter-2) for pyramidal neuron are shown in Additional file 3: Figure S3. To confirm the reduction of GABAergic neurons in SNL-induced anxiety rats, expression of GAD65, GAD67, VGLUT1 and VGLUT2 protein by Western blot were also detected in the CeA. The results showed that expression of both GAD65 (SNL 0.93 ± 0.06 *versus* Sham 1.14 ± 0.05 and Naïve 1.20 ± 0.09, p < 0.05, Figure 8B) and GAD67 (SNL 0.85 ± 0.05 *versus* Sham 1.12 ± 0.07 and Naïve 1.29 ± 0.08, p < 0.01, p < 0.001) were significantly decreased in SNL rats compared to naïve and sham-operated rats (Figure 8C). In contrast, expression of VGLUT1 was increased significantly in SNL-induced anxiety rats (1.27 ± 0.12) compared to naïve (0.88 ± 0.05, p < 0.05) and sham-operated (0.91 ± 0.06, p < 0.05) rats (Figure 8D). Unexpectedly, no significant alteration of VGLUT2 was found in SNL-induced anxiety rats (p > 0.05, Figure 8E). Moreover, immunohistochemical staining showed that the mean optical density of GAD65-positive cells was also decreased prominently in SNL rats (0.19 ± 0.01) compared to naïve (0.25 ± 0.02, p < 0.05) and sham (0.26 ± 0.02, p < 0.05) rats (Additional file 4: Figure S4). Taken together, we here provide direct evidence showing the reduction of GABAergic neurons in the CeA in SNL-induced anxiety rats, which probably contributes to the hypersensitivity of CeA neurons.

**Figure 9:**
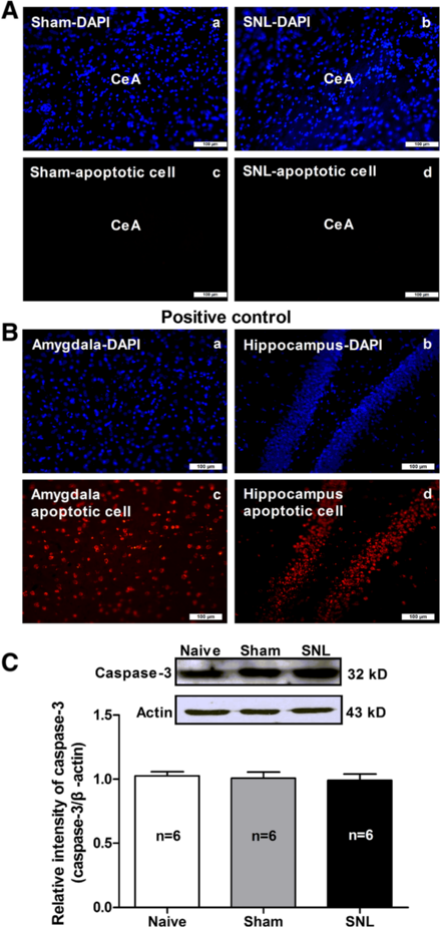
**Detection of apoptosis in the CeA nucleus using TUNEL staining and caspase-3 western blot. (A)**: TUNEL staining. (a) and (b): DAPI staining for marking the nucleus of cells (blue); (c) and (d): TUNEL staining for marking the apoptotic cells (red). Note that not any legible apoptotic cells are observed on the CeA tissue either in SNL or in sham-operated rats. Scale bar = 100 μm, n = 9 tissues from 3 rats/group. **(B)**: positive apoptotic cells control. The amygdala and hippocampal tissues are treated with DNase I recombinant (1000 U/ml) to induce positive apoptotic cells. (a) and (b): DAPI staining for marking the nucleus of cells (blue); (c) and (d): TUNEL staining for marking the apoptotic cells (red). Note that after cells were treated by DNase I, a large numbers of legible apoptotic cells are found in the amygdala and the hippocampal tissues. Scale bar = 100 μm, n = 9 tissues from 3 rats. **(C)**: Western blot assay of caspase-3 expression in the CeA nucleus. Upper: representative of Western blot bands; Lower: analysis of the relative intensity of caspase-3. β-actin is used as an internal control. No significant difference is observed on caspase-3 expression in the CeA nucleus among naïve, sham and SNL rats. n = 6/group.

Furthermore, we determined if the reduction of GABAergic neurons in the CeA was resulted from apoptosis of cells by using methods of both TUNEL (Terminal deoxynucleotidyl transferase dUTP nick end labeling) staining to mark apoptotic cells and Western blot to detect caspase-3, a marker of apoptotic protein. As shown in Figure 9A, no apoptotic cells was found in CeA neurons in both SNL and sham-operated rats, whereas as a positive control after treating cells with DNase I, a large numbers of apoptotic cells were detected in the amygdala and the hippocampal tissues (Figure 9B). Accordantly, no significant difference was observed on expression of caspase-3, a marker of apoptotic protein in the CeA among naïve, sham and SNL-induced anxiety rats (p > 0.05, Figure 9C). Together these results with aforementioned findings of increased expression of VGLUT1 in the CeA in SNL-induced anxiety rats, we suggest that the reduction GABAergic neurons in the CeA is probably due to alteration of neuronal properties (e.g. shift from GABAergic neuron to pyramidal neuron) rather than apoptosis of GABAergic cells.

To further explore whether the reduction of GABAergic cells is associated with enhanced excitability of CeA neurons in SNL rats, we compared spontaneous and evoked firing patterns as well as intrinsic electrogenic properties of cells between pyramidal neurons and GABAergic neurons in CeA in naïve rats. We found that in spontaneous firing mode, there were less multispike and silent firing patterns in GABAergic neurons than those in pyramidal neurons, and more interesting, the tonic firing pattern appeared only in GABAergic neurons. As evoked firing mode was concerned, GABAergic neurons showed more excited features because they had higher percentage of early firing patterns than that in pyramidal neurons (Additional file 5: Figure S5-A). Moreover, we found that in contrast to pyramidal neurons, GABAergic neurons exhibited more depolarized rest membrane potential (−58.50 ± 1.86 mV *versus −*65.86 ± 1.92 mV, GABAergic *versus* pyramidal, p < 0.05, Additional file 5: Figure S5-D), more decreased rheobase (16.36 ± 3.64 pA *versus* 31.43 ± 5.12 pA, GABAergic *versus* pyramidal, p < 0.05, Additional file 5: Figure S5-B) and more reduced frequency of action potentials evoke by 2-folds rheobase current (8.78 ± 0.90 *versus* 13.83 ± 1.25, GABAergic *versus* pyramidal, p < 0.01, Additional file 5: Figure S5-C), whereas no significant difference was observed on the latency of action potentials (p > 0.05, Additional file 5: Figure S5-E). These results suggest that GABAergic neurons have strong inhibitory control to the excitability of CeA neurons. That is probably the reason why reduction of GABAergic cells could induce hyperexcitability of CeA neurons.

### Contribution of GABAergic inhibition in CeA neurons to the development of neuropathic pain-related anxiety

**Figure 10:**
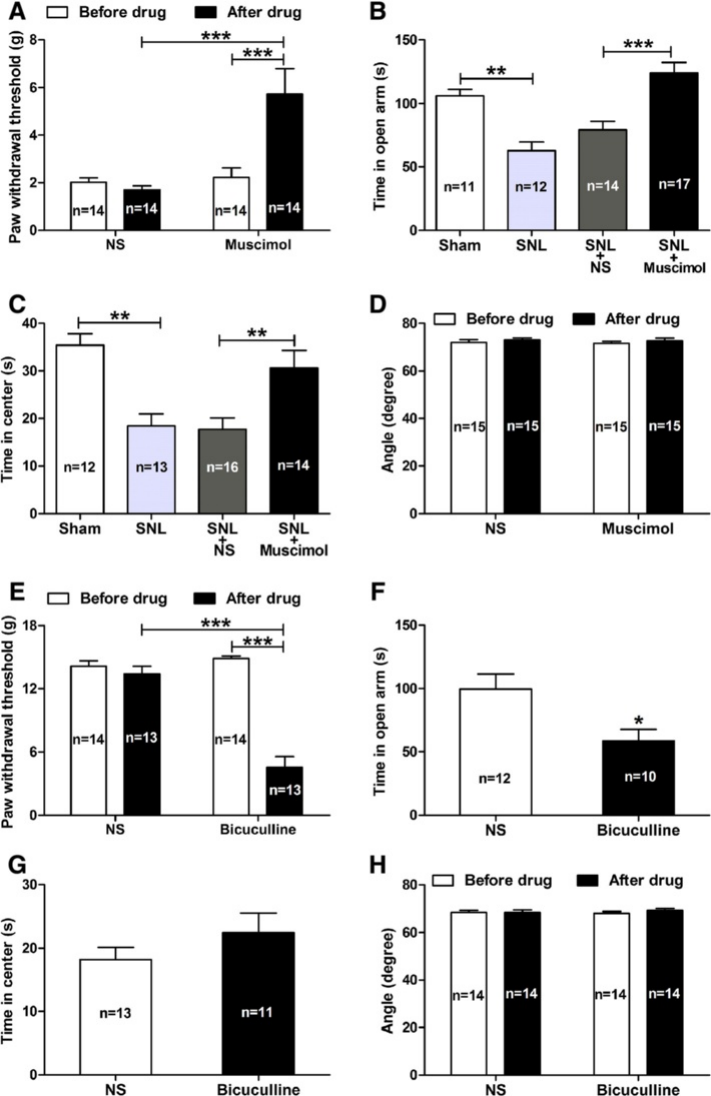
**Effects of intra-CeA administration of muscimol (a GABA**_**A**_ **receptors agonist) or bicuculline (a GABA**_**A**_ **receptors antagonist) on pain behavior and anxiety-like behaviors in rats. (A − D)**: effects of intra-CeA administration of muscimol on pain behaviors **(A)**, anxiety-like behaviors **(B, C)** and locomotor function **(D)** in SNL rats. Note that muscimol (25 ng/μl) significantly rescues the SNL-induced mechanical allodynia (as assessed by PWT, **(A)**) and anxiety-like behaviors (as assessed by elevated plus-maze **(B)** and open-field test **(C)**), but does not affect the locomotor function of rats as assessed by inclined-plate test **(D)**. **p < 0.01, ***p < 0.001, one-way ANOVA, n = 11 − 17/group. **(E − H)**: effects of bicuculline on pain behaviors **(E)**, anxiety-like behaviors **(F, G)** and locomotor function **(H)** in naive rats. Note that bicuculline (25 ng/μl) induces obvious pain allodynia as measured by PWT (**E**, ***p < 0.001, two-way ANOVA, n = 13 − 14/group), and produces anxiety-like behavior as measured by EPM (**F**, *p < 0.05, bicuculline versus NS, two-tailed unpaired t-test, n = 10 − 12/group), but does not affect open-field test (**G**, n = 11 − 13/group). Moreover, intra-CeA administration of bicuculline does not affect locomotor function in naïve rats as measured by inclined-plate test (**H**, n = 14/group).

Next, we investigated whether intra-CeA administration of muscimol, a selective GABA_A_ receptors agonist, could inhibit SNL-induced anxiety-like behaviors in neuropathic rats. We found that both the SNL-induced mechanical allodynia (as assessed by 50% PWT) and anxiety-like behaviors (as assessed by both EPM and open-field test) were significantly inhibited by intra-CeA injection of muscimol (25 ng/μl) [36] to SNL rats. As shown in Figure 10A, the reduced PWT in SNL rats was prominently restored by the treatment of muscimol as compared to NS (muscimol 5.74 ± 1.05 g *versus* NS 1.69 ± 0.17 g, p < 0.001). Similarly, both the reduced time spent in open arm (elevated plus-maze, EPM) and in central area (open-field test, OP test) in SNL rats were also rescued by the treatment of muscimol in contrast to NS (EPM: muscimol 124.2 ± 8.15 s *versus* NS 79.14 ± 6.77 s, p < 0.001; open-field test: muscimol 30.69 ± 3.694 *versus* NS 17.72 ± 2.360, p < 0.01) (Figure 10B and C). As assessed by inclined-plate test, no significant motor dysfunction was found in rats after muscimol administration (p > 0.05, in contrast to NS and pre-drug, respectively, Figure 10D). These results suggest that suppression of CeA neurons excitability via activation of GABAergic neurons likely inhibits the SNL-induced anxiety-like behaviors in rats.

Finally, we determined whether inhibition of GABA_A_ receptors by intra-CeA application of bicuculline, a selective GABA_A_ receptors antagonist, could mimic anxiety-like behaviors in naïve rats. We found that bicuculline (25 ng/μl) not only induced significant mechanical allodynia (as measured by 50% PWT, bicuculline 4.55 ± 1.018 *versus* NS 13.38 ± 0.759, p < 0.001, Figure 10E) but also produced remarkable anxiety-like behavior in naïve rats, because the time spent in open arm (EPM test) was decreased significantly in bicuculline treated rats (NS 99.60 ± 11.99 *versus* bicuculline 58.50 ± 9.24, p < 0.05, Figure 10F), although no statistically difference was found in time spent in central area (open-field test) between bicuculline and NS treated rats (p > 0.05, Figure 10G). Accordantly, as assessed by inclined-plate test, no significant motor dysfunction was found in rats after bicuculline administration (p > 0.05, in contrast to NS and pre-drug, respectively, Figure 10H). These results provide additional evidence showing that enhancement of CeA neurons excitability by GABA_A_ receptors antagonist induces both mechanical allodynia and anxiety-like behavior in normal rats. Together with aforementioned action of muscimol (a selective GABA_A_ receptors agonist) in SNL rats, our present findings suggest that GABAergic inhibition in the CeA plays an important role in the development of nerve injury-evoked anxiety-like behaviors in neuropathic rats.

Location of each drug perfusion was described as black dots in the representative coronal section of CeA (Additional file 6: Figure S6).

## Discussion

In the present study, we disclose a novel mechanism underlying the pathogenesis of nerve injury-evoked anxiety-like behaviors in neuropathic rats. We suggest that reduction of GABAergic inhibition in the CeA contributes to the enhancement of both CeA neurons excitability and output of the amygdala, which subsequently causes the development of neuropathic pain-related anxiety-like behaviors following nerve injury.

### Nerve injury induces pain-related anxiety-like behaviors

We here provide behavioral evidence showing that ligation of L5 spinal nerve in rats produces pain hypersensitivity that is associated with anxiety-like behaviors. We applied both methods of elevated plus-maze and open-field tests to evaluate anxiety-like behaviors in animals, which are widely accepted as behavioral tests for anxiety, and these methods are based on an approach-avoidance conflict generated by fear of open, brightly-lit areas and a drive to explore in new environment [37-40]. Furthermore, by intra-CeA administration of diazepam, a classical anti-anxiety drug that has been widely used in clinic [29], we discovered that diazepam remarkably inhibited the SNL-induced anxiety-like behaviors but did not affect pain behaviors in neuropathic rats. We suggest that nerve injury induces anxiety-like behaviors in neuropathic rats, which are specifically sensitive to anti-anxiety drugs. Additionally, the CeA plays an important role in the development of nerve injury-evoked anxiety-like behaviors. We speculate that these kinds of pain-related anxiety-like behaviors probably emerge at the time point after the development of neuropathic pain. Therefore, selective inhibition of anxiety behaviors can not effectively block already existed pain allodynia in neuropathic rats. In fact, in some mice pain models, it is demonstrated that diazepam has no effect on pain behaviors in different drug doses [15,29]. On the other hand, as a classical analgesic drug, morphine has potent anti-anxiety effects in animals [15,41,42], implying that inhibition of pain behaviors is likely able to prevent the development of anxiety. Taken together, we suggest that the nerve injury-evoked anxiety-like behaviors are pain related.

### Sensitization of CeA neurons in neuropathic pain-related anxiety rats

Despite pain-related anxiety-like behaviors have been reported in several acute and chronic pain models [35,43-46], the role of CeA in pain-induced anxiety is largely unknown. In this study, we present electrophysiological evidence demonstrating that nerve injury produces a significant increase both in burst firing pattern and early firing pattern as well as in spike frequency in CeA neurons (Figure 2 and 3), suggesting a sensitization or hyperexcitability of amygdala output in neuropathic pain-induced anxiety rats. Our current results agree with previous report by Dickenson et al. [47] who have found both the spontaneous neuronal activity and the firing frequency of right CeA neurons are increased dramatically in SNL rats. Accordantly, Neugebauer and colleagues have also observed an increased excitability of CeA neurons in animal models of inflammatory pain [48] and visceral pain [49]. We hence propose that sensitization of amygdala neurons, which leads to the enhancement of CeA output, probably underlies the neuropathic pain-related anxiety. It is well known that the right amygdala plays a dominant role in the modulation of pain processing and related negative emotions [47,50-52]. In animal models of chronic pain, Goncalves et al. [47] have reported that hemispheric lateralization of pain processing in the amygdala is independent of the side of the peripheral injury, and right amygdala shows higher activity than the left [47]. In this study, we mainly focused on the right rather than the left amygdala. Therefore, all examined neurons in our present study were recorded from the right CeA. Additionally, since CeA is a complex nucleus including multiple neuronal types [9-11,53], identifying neuronal types and firing patterns in the CeA is of significant to examine excitabilities of the CeA neurons and their contribution to pain-related anxiety. With regard to classification of firing patterns in CeA neurons, especially under spontaneous recording mode, it is of course that the firing patterns of some neurons can not be classified so distinct. For example, those firing simultaneously mixed with multispike and burst pattern in the same neuron. In our present study, all neurons exhibiting burst firing with or without multispike are defined as burst firing pattern, since the neurons with mixed burst firing are still more hyperexcited than those with pure multispike firing. The aim of our current study is to investigate hyperexcitability of CeA neurons in nerve injury-evoked anxiety-like behaviors, hence mixed burst firing and pure burst firing are merged as one firing pattern in the present study.

As a “hub” in the emotional neural circuit, CeA is the main output of the amygdala, which finally projects to hypothalamus and brain stem to regulate emotional response and motor behaviors [10-12,54]. Therefore, excitability of CeA neurons plays a crucial role in the regulation of affective component of pain. Burst firing as a form of epileptiform activity is proven to be an overexcited firing pattern of neurons [55]. Spike with short latency in early-firing pattern is another form of excited neuronal firing. Spike-frequency adapting occurs in instantaneous depolarization in voltage threshold of action potential. In this condition, reduced sodium current availability results in the firing adapting [56]. However, spike adapting reduces the number of spikes and the output of amygdala, which implies neuronal homeostatic response to hyperexcitability of the CeA [57]. Since neuronal firing is regarded as output of neurons, with which amygdala transmits neuronal information to other brain regions in emotional neural circuit. Our current results imply the occurrence of enhanced output of the amygdala in SNL-induced anxiety rats, which may be a key factor for the pathogenesis of neuropathic pain-related anxiety.

### Roles of burst firing pattern in development of neuropathic pain-related anxiety

A large numbers of evidence support the notion that the burst firing pattern means hyperexcited neuronal output in the brain [58-60]. The generation of burst firing has been widely studied in the field of sleep, epilepsy and Parkinson’s disease. It is reported that sleep spindle as one type of several rhythmic brain waves is mediated by T-type calcium channel-elicited burst firing in excitatory thalamocortical neurons, which is an important mechanism for cortical-thalamo-cortical circuit [61]. In rewarding behaviors, burst of spikes is increased in response to novel salient stimuli, and is accompanied by transient increment in dopamine levels [62,63]. Therefore, burst firing of neurons plays a key role in the regulation of spike output and thereby relating behaviors expression in neuropathic conditions. In the present study, we found that the pattern of burst firing but not tonic firing was increased significantly in CeA neurons in SNL rats (Figure 2), indicating the enhancement of amygdala output which finally regulates the affective component of neuropathic pain.

Here, a key question should be elucidated, i.e. what factors contribute to increased burst-firing pattern in SNL-induced anxiety rats? Sub-threshold membrane potential oscillation, an important intrinsic electrogenic factor that is related to action potential firing and neuronal excitability, has been reported in dorsal root ganglia, olfactory bulb, thalamus and entorhinal cortex [55,61,64,65]. In these neurons, action potential firing can be elicited by sinusoidal oscillation in membrane potential. Once peaks of sub-threshold oscillation reaches threshold of action potential, then spike arises [66]. Moreover, the authors have found that the oscillation sinusoidal reaches threshold intermittently, then yields slow irregular firing in normal conditions [66-69]. Thus, we speculate that subthreshold oscillation likely contributes to increased burst firing in neuropathic conditions. Our present findings showed that in neuropathic pain-induced anxiety rats, amplitude, but not frequency of membrane potential oscillation was increased prominently (Figure 5), which may underlie the increased burst-firing pattern in SNL rats. Another intrinsic electrogenic factor is after depolarization potential (ADP), which is also implicated to the formation of burst firing [70]. Actually, we found that in CeA neurons, the shape of action potentials (AP) was variable dependent on the characteristics of ADP, after-hyperpolarization (AHP) and depolarization before reaching peak of spike (Figure 4). Moreover, the type of AP with obvious ADP accounted for the main population of CeA neurons under spontaneous firing state. Because ADP is close associated with burst firing and it plays a key role in the maintaining and termination of burst spike [55], thus the electrogenic properties of neurons with ADP were also examined in our current study. We present evidence demonstrating that amplitude, but not frequency of ADP was significantly increased in SNL rats. Taken together, these findings suggest that both the increased ADP and the membrane potential oscillation may underlie the increased burst-firing pattern in SNL rats, which as an indicator of enhanced amygdala output likely contributes to the development of neuropathic pain-induced anxiety.

### Reduction of GABAergic inhibition in the CeA neurons contributes to the development of neuropathic pain-related anxiety

Several studies have shown that pallidal GABAergic neurons have a tonic control to the firing pattern of glutamatergic subthalamic nucleus (STN). For example, GABA_A_ receptors agonist can specifically reduce the firing rate of the STN neurons whereas GABA_A_ receptors antagonist strongly reinforces the spontaneous bursting pattern into a marked one with instantaneous frequency reaching 500–600 Hz [71,72]. In consistent with these findings, our present study revealed that in contrast to pyramidal neurons, GABAergic neurons were featured with more depolarized RMP, more lower rheobase and more higher frequency evoked by 2-folds rheobase current application (Additional file 5: Figure S5), indicating the tonic GABAergic inhibition in the CeA, which is proposed to be responsible for regulating the firing patterns and the intrinsic excitability of CeA neurons. In fact, we found that GABA_A_ receptors antagonist bicuculline could remarkably enhance the membrane potential oscillation, increase the evoked spike frequency, and raise the adapting-firing pattern under amygdala slice recording (Additional file 7: Figure S7). In addition, by using single-cell PCR, Western blot and immunohistochemical techniques, we further observed that expression of protein markers of GABAergic neurons, GAD65 and GAD67, were decreased significantly in the CeA in SNL rats (Figure 8 and Additional file 4: Figure S4), suggesting the reduction of GABAergic inhibition under neuropathic condition. Furthermore, we provide additional evidence demonstrating that the reduction GABAergic neurons in the CeA is probably due to alteration of neuronal properties (e.g., shift from GABAergic neuron to pyramidal neuron) rather than apoptosis of GABAergic cells, because not any apoptotic cells or upregulation of caspase-3 (a marker of apoptotic protein) were found in the CeA neurons in SNL-induced anxiety rats, whereas expression of VGLUT1 was significantly increased.

To further clarify whether the reduction of GABAergic inhibition contributes to neuropathic pain-related anxiety-like behaviors, we first examined effects of muscimol (intra-CeA administration), a selective GABA_A_ receptors agonist on neuropathic pain-induced anxiety in SNL rats. We here provide pharmacological behavioral evidence showing that suppression of CeA neurons excitability via activation of GABAergic neurons inhibits the SNL-induced anxiety-like behaviors in neuropathic pain rats. On the contrary, intra-CeA administration of bicuculline, a selective GABAA receptors antagonist produces both pain hypersensitivity and anxiety-like behavior in normal rats, indicating that enhancement of CeA neurons excitability via reduction of GABAergic inhibition results in pain-related anxiety. It is well established that anxiety disorders and major depression share a GABAergic deficit as common pathophysiological mechanism [73], and GABA_A_ receptor is involved in the modulation of anxiety and depression-like behaviors [73-76]. It is thus that reduction of GABAergic inhibition in the CeA plays an important role in the development of nerve injury-evoked anxiety-like behaviors in neuropathic rats.

In conclusion, our present study suggests that the decreased GABAergic inhibition may be responsible for regulating the firing patterns and the hyperexcitability of CeA neurons, which likely underlie the enhanced output of amygdala and the neuropathic pain-related anxiety in SNL rats.

## Material and methods

### Animals

Male Sprague – Dawley rats weighing 150–180 g at the beginning of the experiment were provided by the Department of Experimental Animals Sciences, Peking University Health Science Center. The rats were housed in separated cages with free access to food and water. The room temperature was kept at 24 ± 1°C under natural light – dark cycle. All animal procedures were carried out in accordance with the guidelines of the International Association for the Study of pain [77] and were approved by the Animal Care and Use Committee of Peking University.

### Spinal nerve ligation

Under general anesthesia with chloral hydrate (0.3 g/kg, intraperitoneally, i.p.), the left lumbar 5 (L5) spinal nerves distal to the dorsal root ganglia were tightly ligated with 4–0 silk sutures as described by Kim and Chung [78]. In control animals, sham surgery with identical procedure except for ligation of the L5 spinal nerves was received. Any rats exhibiting motor deficiency or lack of tactile allodynia were excluded from the study.

### Behavioral studies

#### Assessment of mechanical allodynia

Mechanical allodynia, as a behavioral sign of neuropathic pain, was assessed by measuring 50% paw withdrawal threshold (PWT) as described in our previous reports [79,80]. The 50% PWT in response to a series of von Frey filaments (Stoelting, Wood Dale, IL, USA) was determined by the Up and Down method [81]. The rat was placed on a metal mesh floor covered with an inverted clear plastic cage (18 × 8 × 8 cm) and allowed a 20-min period for habituation. Eight von Frey filaments with approximately equal logarithmic incremental (0.224) bending forces were chosen (0.41, 0.70, 1.20, 2.00, 3.63, 5.50, 8.50, and 15.10 g). Each trial started with a von Frey force of 2.00 g delivered perpendicularly to the plantar surface of the left hindpaw for about 2–3 s. An abrupt withdrawal of the foot during stimulation or immediately after the removal of the hair was recorded as a positive response. Whenever there was a positive or negative response, the next weaker or stronger filament was applied, respectively. This procedure was done until six stimuli after the first change in response had been observed. The 50% PWT was calculated using the following formula: PWT = 10^[Xf + kδ]^, where X_f_ is the value of the final von Frey filament used (in log units), wk is a value measured from the pattern of positive/negative responses, and δ = 0.224, which is the average interval (in log units) between the von Frey filaments [82]. If an animal responded to the lowest von Frey filament, a value of 0.25 g was assigned. If an animal did not respond to the highest von Frey filament, the value was recorded as 15.0 g. Testing sessions were performed on the day before surgery and day 7 after SNL or sham surgery. In rats, mechanical allodynia is assessed by measuring ipsilateral PWT in response to von Frey filaments, and an allodynic rat is defined as that the 50% PWT was less than 4.0 g, i.e. withdrawal in response to non-noxious tactile stimulus [83].

#### Elevated plus-maze test and open-field test

Anxiety-like behavior was evaluated by elevated plus-maze (EPM) test [84] through an apparatus consisting of two open and two closed arms (48 × 8 × 40 cm each arm) (Shanghai Mobiledatum information Technology Co., Ltd, Shanghai, China). Each rat was placed in the centre of the elevated plus-maze facing one of the open arms, and the time spent in the open or closed arms was recorded during a 5-min test period. The elevated plus-maze was carefully cleaned with 10% ethanol before each animal was placed on the equipment.

The apparatus for the open-field test [37] was a box (100 × 100 × 50 cm) made of opaque materials (Shanghai Mobiledatum information Technology Co., Ltd, Shanghai, China). The open-field arena was partitioned into 25 equal-size squares. The test was conducted in a quiet room in the morning (8:00 – 12:00 a.m.). Each rat was placed in the center of the area and its behavior was recorded for 5 min. The time spent in the center was an index of anxiety-like behavior. The open-field was cleaned after each test.

The behavioral procedures of both EPM test and open-field test were kept in video recording for further analysis of behavioral results with smart video tracking software. All of behavioral tests were operated under single-blind experiment.

#### Assessment of locomotor function

Inclined-plate test was used for the assessment of locomotor function. The rat was placed crosswise to the long axis of an inclined plate. The initial angle of the inclined plate was 50 degrees. The angle was then adjusted in 5-degree increments. The maximum angle of the plate on which the rat maintained its body position for 5 s without falling was determined according to the method reported by Rivlin and Tator [84].

#### Stereotaxic surgery and intra-CeA microinjection

Under general anesthesia with pentobarbital sodium (0.5 g/kg, i.p.), rats (250–300 g) were placed in a stereotaxic frame (Kopf Instruments, Tujunga, CA, USA) with the incisor bar adjusted to achieve a flat skull position relative to lambda and bregma. Using bregma as the reference point, guiding cannulas (RWD Life Science) were implanted bilaterally (anterior-posterior (AP) -2.3 mm; lateral (L) ± 4.5 mm; dorsal-ventral (DV) -7.0 mm) according to coordinates obtained from Paxinos and Watson (1997) [79]. The bilateral cannulas were permanently secured to the skull surface using dental acrylic anchored with 4 skull screws. Following surgery, animals were allowed a total recovery period of 5 days. Simultaneous bilateral microinjection of drugs or vehicle into the amygdala in conscious rats were made using 2-μl Hamilton syringes connected to infusion a cannula (RWD Life Science) via PE-50 tubing. The infusion cannula projected 1.0 mm below the tip of the guiding cannula. A total of 0.5 μl solution was *in vivo* injected into each side of the CeA over a 60-s time period, with the infusion cannula left in place for an additional 60 s to prevent backflow of drug up to the guiding cannula.

### Electrophysiological studies

#### Preparation of the amygdala slices

After decapitated, the rat brain was quickly dissected out and blocked in cold (4°C) artificial CSF (ACSF) containing (in mM): 125 NaCl, 2.5 KCl, 2 CaCl_2_, 1 MgCl_2_, 25 NaHCO_3_, 1.25 NaH_2_PO_4_ and 25 Glucose. The ACSF was oxygenated and equilibrated to pH 7.4 with a mixture of 95% O_2_ and 5% CO_2_. Coronal brain slices (300 μm) were prepared using a Vibroslice (Leica Instruments, Heidelburg, Germany). After incubation in ACSF at 34°C for at least 0.5 h, a single brain slice was transferred to the recording chamber and submerged in ACSF (31 ± 1°C) for superfusing the slice at ~2 ml/min.

#### Whole-cell patch-clamp recording

Whole-cell current-clamp recording was performed at room temperature using a Multiclamp 700B amplifier and Clampex software (Molecular Device, Sunnyvale, CA USA). Patch pipettes were pulled from borosilicate glass capillaries with a tip resistance of 2–5 MΩ when filled with internal solution containing (in mM) 140 KCl, 1 CaCl_2_, 2 MgCl_2_, 11 EGTA and 10 HEPES, adjusted to pH 7.2 with KOH. The external solution contained (in mM) 125 NaCl, 2.5 KCl, 2 CaCl_2_, 1 MgCl_2_, 25 NaHCO_3_, 1.25 NaH_2_PO_4_ and 25 Glucose, adjusted pH 7.4 with NaOH. Spontaneous firing and evoked action potentials (APs) were measured with pipette and membrane capacitance cancelation, filtered at 2 kHz and digitized at 10 kHz. Series resistance was compensated at 70–90%.

Under current-clamp mode, the spontaneous firing of CeA neurons in brain slice was first recorded in gap-free mode at the holding current of 0 pA, lasted for 1 min. This protocol was aim to classify the firing patterns of CeA neurons. The following parameters were measured under spontaneous firing recording mode: ratio of firing pattern in each group, amplitude and frequency of after depolarization potential (ADP) as well as amplitude and frequency of membrane potential oscillation. To further investigate the relationship between injection current and spike frequency in CeA neurons, cell was held at 0 pA, and a series of 600-ms depolarizing current pulses in 10 pA increment from 0 pA to 110 pA were delivered to evoke the cell generating action potentials. A hyperpolarizing current pulse in 600 ms, −100 pA was delivered to measure membrane input resistance (R_in_), which was assessed from the value of the evoked membrane potential divided by the injected hyperpolarizing current (−100 pA). A single 600 ms, 300 pA depolarizing current was injected into cell to measure the ratio of adapting firing and frequency change in each groups. The following parameters were measured under evoked discharges recording mode: baseline resting membrane potential (RMP), frequency of spontaneous firing, numbers of evoked AP, amplitude and duration of AP, rise (or fall) rate of AP, threshold potential (TP), rheobase for evoked the 1^st^ AP (I_th_), inter-spike interval (ISI) as well as the first and second amplitude of APs.

### Single-cell reverse transcriptase PCR

For single-cell RT-PCR experiments, the patch pipettes were filled with 6 μl of autoclaved internal RT-PCR solution containing (in mM): 140 KCl, 5 HEPES, 5 EGTA and 3 MgCl_2_ (pH 7.3). At the end of recording (<15 min), the cell contents (including the nucleus, in most cases) were aspirated as completely as possible into the patch pipette under visual control (63× objective + 2 − 4× zoom) by application of gentle negative pressure. Cells were only analyzed further when the whole-cell configuration remained stable throughout the harvesting procedure. Pipettes were then quickly removed from the cell, washed twice through the solution interface, and the pipette contents were expelled immediately into a 0.5-ml test tube containing the contents for reverse transcription. First strand cDNA was synthesized for 1 h at 37°C in a total reaction volume of 10 μl containing random hexamer primers (Takara Bio Inc., otsu, Japan, final concentration 5 μM), dithiothreitol (DTT, final concentration 10 mM), the four deoxyribonucleotide triphosphates (dNTPs, final concentration 0.5 mM each) (Takara Bio Inc.), 20 U of ribonuclease inhibitor (Takara Bio Inc.) and 100 U of reverse transcriptase (Takara Bio Inc.). The single-cell cDNA was kept at −80°C until PCR amplification.

Following reverse transcription, the cDNAs for Vesicular glutamate transporter I (VGLUT1), Vesicular glutamate II (VGLUT2), Glutamic acid decarboxylase 65 (GAD65), Glutamic acid decarboxylase 67 (GAD67) and β-actin were amplified simultaneously in a multiplex PCR using the following set of primers (from 5′ to 3′): VGLUT 1, forward GGCCCCTCCCTTAGAACG, reverse CCTCCGATGGGTACGATGATA; VGLUT 2, forward GAGCCCCGCAAAGCATC, reverse CTCGGGGCAATATCCAAGTG; GAD65, forward CCTTTCCTGGTGAGTGCCACAGCTGGAACC, reverse TTTGAGAGGCGGCTCATTCTCTCTTCATTG; GAD67, forward ACCCTGGTGCCCGCTTCC, reverse TATTGGTATTGGCAGTTGATGTC; β-actin, forward AGCCATGTACGTAGCCATCC, reverse GCCATCTCTTGCTCGAAGTC. First multiple-PCR was performed as hot start in a final volume of 100 μl containing the 10 μl reverse transcription reaction, 100 pmol of each primer, 0.2 mM each dNTP (Takara), 1.8 mM MgCl_2_, 50 mM KCl, 20 Mm Tris–HCl (Ph 8.4) and 3.5 U of Taq polymerase (Takara) in a Perkin Elmer Thermal Cycler 480C with the following cycling protocol: after 3 min at 94°C, 35 cycles (94°C, 30 s; 58°C, 60 s; 72°C, 3 min) of PCR were performed followed by a final elongation period of 7 min at 72°C. The nested-PCR amplifications were carried out in eight individual reactions, in each case with 2.5 μl of the first PCR product under similar conditions with the following modifications: 50 pmol of each primer, 2.5 U of Taq polymerase, 1.5 mM MgCl_2_ and a shorter extension time using the following primer pairs: VGLUT1, forward CCTTTTGCGGTTCCTATGC, reverse AATGTATTTGCGCTCCTC CTC; VGLUT2, forward TCAAGACCCCATGGAGGAAGT, reverse GTTCATGATCTTTCGCACTGTAG; GAD65, forward GAGCCCCGCAAAGCATC, reverse CTCGGGGCAATATCCAAGTG; GAD67, forward GGACTTCCACCACCCACAC, reverse CTAAACCAATGATATCCAAACCAG; β-actin, forward GAGCCCCGCAAAGCATC, reverse CTCGGGGCAATATCCAAGTG. To investigate the presence and size of the amplified fragments, 30 μl aliquots of PCR products were separated and visualized in an ethidium bromide-stained agarose gel (2%) by electrophoresis. The predicted sizes of the PCR products were (bp) 311 (VGLUT1), 419 (VGLUT2), 599 (GAD65), 401 (GAD67) and 294 β-actin. All individual PCR products were verified several times (n > 3) by direct sequencing or by subcloning and sequencing.

### Immunohistochemistry

Deeply anesthetized rat was intracardially perfused with 300 ml of 0.1 M phosphate buffer (PB) followed by 300 ml of 4% paraformaldehyde (in 0.1 M PB, pH 7.4). The brain was quickly removed, postfixed in the same fixative solution for 6 hours and then was cryoprotected in 30% sucrose (in 0.1 M PB). Several days later, the tissues were sectioned in 30-μm thickness on a cryostat. After blocking in 10% normal goat serum for 1 hour, the sections were incubated with affinity-purified rabbit antibody to GAD65 (1:100, millipore, USA) at 4°C overnight. Control sections were processed without the addition of primary antibody. The sections were then washed in 0.1 M phosphate-buffered saline (PBS) and incubated with biotinylated goat anti-rabbit immunoglobulin G (1:300, Invitrogen, Carlsbad, CA, USA) at 37°C for 30 minutes. After several washes in PBS, the sections were incubated in the avid in-biotin-peroxidase complex (1:300, ABC Elite, Invitrogen) at 37°C for 45 minutes. The horseradish peroxidase reaction was developed in 0.1 M Tris-buffered saline (pH 7.4) containing 0.05% 3,3P-diaminobenzidine and 0.01% H_2_O_2_. The sections were then dehydrated and coverslipped. For relative quantification of immunoreactivity, a computer-assisted image analyzer (Image Pro Plus, version 6.0, Media Cybernetics, Silver Spring, MD) was used at a magnification of × 20 and × 5 Image which was acquired using a cooled CCD camera (Spot 2; Diagnostic Instruments, Sterling Heights, Mi), mounted on a Leica DMI 3000B microscope (Leica, Wetzlar, Germany). The ratio of GAD65-positive cells compared with the total neuronal profiles and the average optical intensity of GAD65-positive in amygdala slice were calculated in a blind fashion to identify any relative changes in expression of GAD65 in the amygdala. The average intensity was defined as the difference between average gray value (mean integrated optical density) within each amygdala section and its background. Each threshold density in every slice was determined for positive immunoreactivity above background, and the thresholds were kept constant for all samples. Neurons that had densities higher than a threshold density were considered to show positive expression. A mean value was calculated for each amygdala slice in each brain, and all individual values for each amygdala slice were averaged to provide a single mean ± SEM.

### Western blot assays

Under deeply anesthetized with 10% chloral hydrate (0.3 g/kg, i.p.), the rat brain was extracted quickly. The right CeA tissue was obtained and homogenized with RIPA lysis buffer (Beyotime Biotechnology, Haimen, Jiangsu Province, China). The protein concentrations of all of the samples were determined using the BCA assay kit (Pierce, Rockford, IL, USA). The equivalent of 50 μg total protein was added to the loading buffer containing 2% sodium dodecyl sulfate (SDS), 100 mM Dithiothreitol (DTT), 10% glycerol, 0.25% bromophenol blue, and boiled for 5 min. Proteins were separated on a 10% acrylamide resolving gel and then transferred to a polyvinylidene difluoride filters membrane (Bio-Rad, Hercules, CA, USA). After blocking with 5% nonfat milk in Tris-buffered saline and Tween (TBST, 20 mM Tris–HCl, pH 7.5, 150 mM NaCl, and 0.05% Tween-20) for 60 min at room temperature, the membranes were respectively incubated with the following primary antibodies at 4°C overnight: mouse anti-GAD65 (1:1000, Abcam Inc., Cambridge, MA, USA); mouse anti-GAD67 (1:1000, EMD Millipore, Darmstadt, Germany); rabbit anti-VGLUT1 (1: 200, Abcam); goat-VGLUT2 (1:200, Abcam) or rabbit anti-caspase-3 (1:100, Santa Cruz Biotech, Dallas, Texas, USA). The blots were washed in TBST and then incubated in horseradish peroxidase–conjugated secondary antibody (1:1000, goat anti-rabbit or mouse or donkey anti-goat, Jackson, Bar Harbor, Maine, USA). Protein bands were visualized using an enhanced chemiluminescence detection kit (ECL, Santa Cruz Biotechnology, Inc.) followed by autoradiography using Hyperfilm MP (Santa Cruz Biotechnology, Inc.). Band intensity was quantified using Quantity One software (version 4.0.3) from Bio-Rad (Hercules, CA, USA).

### TUNEL (Terminal deoxynucleotidyl transferase dUTP nick end labeling) staining

Deeply anesthetized rat was intracardially perfused with 50 ml of 0.1 M PB followed by 500 ml of 4% paraformaldehyde (in 0.1 M PB, pH 7.4). The brain was quickly removed, post-fixed in the same fixative solution for 8 hours, and then was dehydrated and cryoprotected in 20% sucrose (in 0.1 M PB) and 30% sucrose solution orderly. Several days later, the tissues were sectioned in a 20-μm thickness on a cryostat. Tissues were washed three times with PBS and then permibealized in 0.1 M citrate buffer (PH 6) in 750 W microwave irradation for 1 min. After cooled rapidly in double distilled water, tissues were transferred into PBS solutions followed by TUNEL labeling. Detection kit, TMR red was carried out according to the manufacturer’s instruction (Roche, Indianapolis, Indiana, USA).

As a positive control for marking apoptotic cells, tissues were pre-treated by DNase I recombinant (Roche) (1000 U/ml in 50 mM Tris–HCl, PH 7.5 and 1 mg/ml bovine serum albumin) for 10 min at 15 to 20°C to DNA strand breaks, prior to labeling procedures.

### Drugs and administration

Muscimol (5-aminomethyl-3-hydroxyisoxazole) and (−)-bicuculline methiodide [R-(R*,S*)]-5-(6,8-Dihydro-8-oxofuro[3,4-e]-1,3-benzodioxol-6-yl)-5,6,7,8-tetrahydro-6,6-dimethyl-1,3-dioxolo[4,5-g] isoquinolinium iodide were purchased from Tocris Bioscience. Both drugs were dissolved in sterile saline solution at a final concentration 25 ng/μl and a total of 0.5 μl solution was in vivo administered into each side of the CeA by microinjection at 30 min prior to behavioral test. Doses of muscimol and bicuculline used in the present study were determined from previous studies in which these compounds were injected directly into the amygdala [36,80,85]. Diazepam was purchased from Sigma-Aldrich (Saint Louis, MO, USA) and was dissolved in methanol in a final concentration of 1 μg/μl. A total of 1 μl solution was in vivo administered into each side of the CeA by microinjection twice in a 2-min interval at 30 min before behavioral test according to the methods described in previous report [86].

### Statistical analysis

Statistical analysis was performed with GraphPad Prism 5 for Windows (GraphPad Software, Inc, La Jolla, CA, USA). All data are expressed as mean ± SEM. Two-tailed unpaired t-test was used for the comparison of the mean values between two groups. One-way analysis of variance (ANOVA) followed by Dunnett’s multiple comparison test or two-way ANOVA followed by Bonferroni post-hoc test was used for multiple comparison. Differences with p < 0.05 were considered statistically significant.

## Additional files

Additional file 1: Figure S1. Effects of intra-CeA administration of diazepam (a classical anti-anxiety drug) on SNL-induced pain behaviors in rats. Note that intra-CeA administration of diazepam (2 μg/μl) does not affect the SNL-induced pain allodynia as measured by paw withdrawal threshold. P > 0.05, two-way ANOVA, n = 9 NS and 11 diazepam. Additional file 2: Figure S2. Some electrogenic properties of CeA neurons are not altered in SNL-induced anxiety rats. (A): maximal rise time. (B): maximal decay time. (C): rise slope. (D): input resistance (R_in_). (E): decay slope. All these intrinsic electrogenic parameters of action potentials (AP) including maximal rise/decay time and rise/decay slope are measured from the 1^st^ AP evoked by a series of depolarizing step currents (duration 600 ms, increment 10 pA) from 0 pA to 110 pA, while the R_in_ is evoked by a hyperpolarizing current pulse (600 ms, −100 pA). Note that no statistically difference is observed on these intrinsic electrogenic properties in the CeA neuron among naïve, sham and SNL rats. P > 0.05, one-way ANOVA, n = 11 − 27/group. All recorded cells in each group are obtained from animals of naïve (5 rats); sham (4 rats) and SNL (6 rats), respectively, except for the summary of R_in_ is obtained from animals of naïve (6 rats); sham (5 rats) and SNL (7 rats). Additional file 3: Figure S3. Detection of VGLUT1, VGLUT2, GAD65 and GAD67 mRNA expression. (A): identification of the validity of the following primer pairs including VGLUT1, VGLUT2, GAD65 and GAD67 (for multiplex-PCR), and VGLUT1′, VGLUT2′, GAD65′ and GAD67′ (for rest-PCR) in hippocampal tissues by reverse transcriptase-PCR. (B): representative of single cell transcriptase-PCR analysis for the discrimination of pyramidal and GABAergic neuron. Sample sequence (from right to left): VLUT1, VLUT2, GAD65 and GAD67. Additional file 4: Figure S4. Reduction of GAD65 expression in SNL-induced anxiety rats. (A − C): examples represent the immunohistochemical staining of GAD65 in the CeA in naïve (A), sham (B) and SNL (C) rats, respectively. (D): summary of mean optical density of GAD65 expression. Note that the mean optical density values of GAD65 expression in the CeA are decreased significantly after in SNL rats compared to naïve and sham rats. *p < 0.05, one-way ANOVA, n = 9 tissues obtained from 3 rats/group. Additional file 5: Figure S5. Comparison of electrogenic properties between GABAergic and pyramidal neurons in naïve rats. (A): comparison of firing patterns between pyramidal and GABAergic neurons. Left panel: distribution of spontaneous firing patterns in pyramidal and GABAergic neurons. Right panel: distribution of evoked firing patterns in pyramidal and GABAergic neurons. Note that GABAergic neurons show more excited features than pyramidal neurons. (B − E): comparison of rheobase, spike frequency (evoked by 600 ms, 100 pA current pulse), rest membrane potentials (RMP) and the 1^st^ spike latency (evoked by 600 ms, 100 pA current pulse) between pyramidal and GABAergic neurons. Note that the rheobase (B) and spike frequency (C) are much lower, and the RMP (D) is more depolarized in GABAergic neurons than those in pyramidal neurons. No statistically difference is observed on the 1^st^ spike latency between pyramidal and GABAergic neurons (E). *p < 0.05, **p < 0.01, pyramindal *versus* GABAergic neurons, two-tailed unpaired t-test, n = 14 pyramidal cells from 5 rats and 11 GABAergic cells from 4 rats, respectively. Additional file 6: Figure S6. Histological representation of bilateral injection sites upper the proximity of the central nucleus of the amygdala (CeA). (A − B): representative Nissl staining of left (A) and right (B) CeA after the behavioral test. Scale bar: 500 μm. Cavity areas upper the CeA are the injection sites. (C): for all tested animals, the tip of each injection cannula is represented by a black dot, and the localization described within coronal sections of the rat brain is conducted according to the atlas of Paxinos and Watson (1997) [79]. Additional file 7: Figure S7. Effects of bicuculline, a selective GABA_A_ receptors antagonist, on membrane potential oscillation as well as spike frequency and firing patterns of CeA neurons. (A): examples represent for sub-threshold membrane potential oscillation before and after perfusion of bicuculline(10 μM) in spontaneous discharges recording mode. Note that the sub-threshold membrane potential oscillation is obviously increased after perfusion of bicuculline. (B − C): effect of bicuculline (10 μM) on spike frequency (B) and firing patterns (C) in the CeA neurons. Note that perfusion of bicuculline causes an increase in the frequency of action potentials evoked by a depolarizing current pulse (600 ms, 100 pA) and a shift of firing pattern from no-adapting firing to adapting firing pattern in the CeA neurons. n = 5 cells obtained from 3 rats.

## Abbreviations

LA/BLA: Lateral/basal lateral amygdale
CeA: Central nucleus of the amygdale
APs: Action potentials
PWT: Paw withdrawal threshold
SNL: Spinal nerve ligation
EPM: Elevated plus-maze
NS: Normal saline
CNS: Central nervous system
ADP: After depolarized potential
AHP: After hyperpolarized potential
RMP: Rest membrane potential
R_in_: Input resistance
ISI: Inter-spike interval
TUNEL: Terminal deoxynucleotidyl transferase dUTP nick end labeling
i.p.: Intraperitoneally
AP: Anterior-posterior
DV: Dorsal-ventral
ACSF: Artificial cerebral spinal fluid
TP: Threshold potential
DTT: Dithiothreitol
dNTPs: Deoxyribonucleotide triphosphates
VGLUT 1: Vesicular glutamate transporter I
VGLUT 2: Vesicular glutamate II
GAD65: Glutamic acid decarboxylase 65
GAD67: Glutamic acid decarboxylase 67
PB: Phosphate buffer
PBS: Phosphate-buffered saline
SDS: Sodium dodecyl sulfate
DTT: Dithiothreitol
TBST: Tris-buffered saline and Tween
